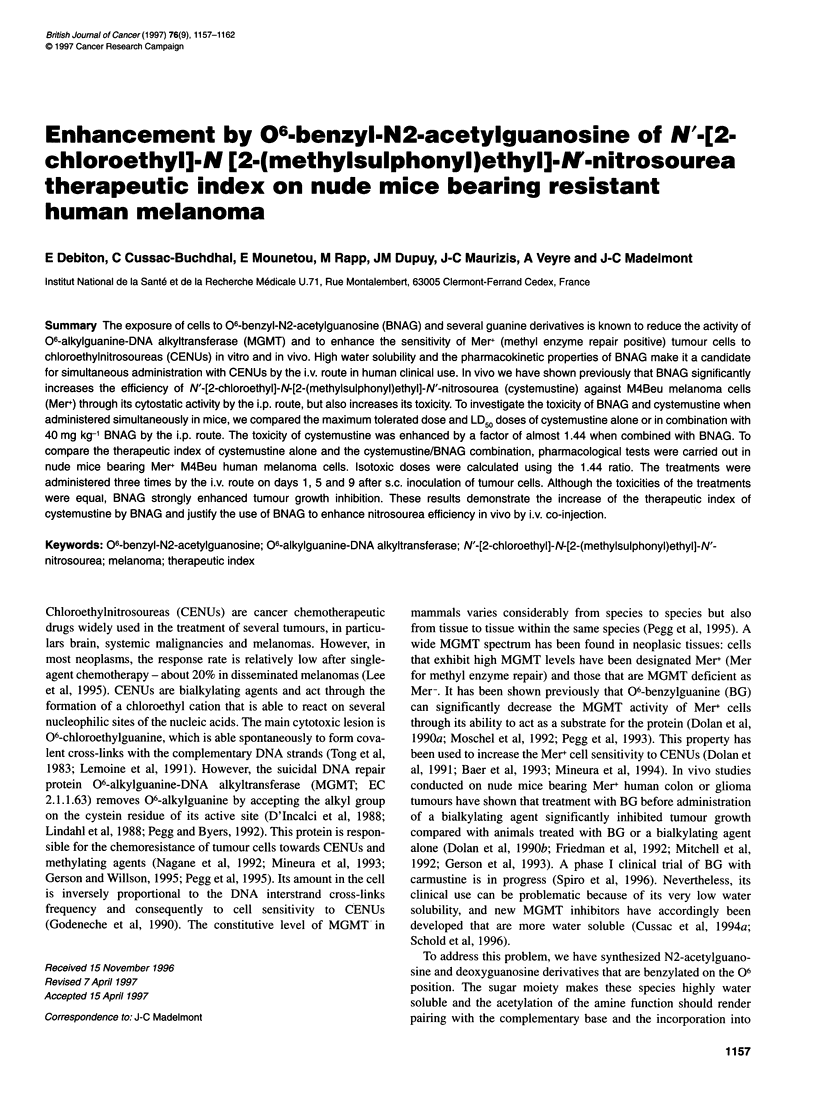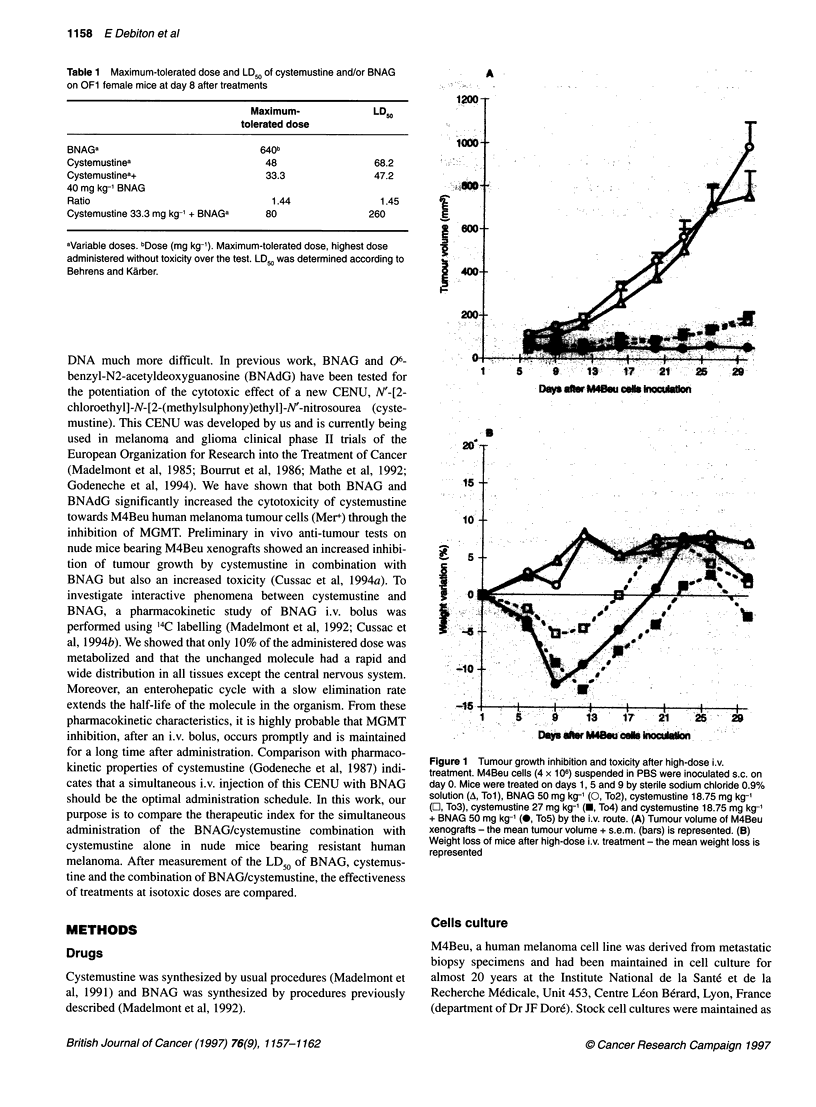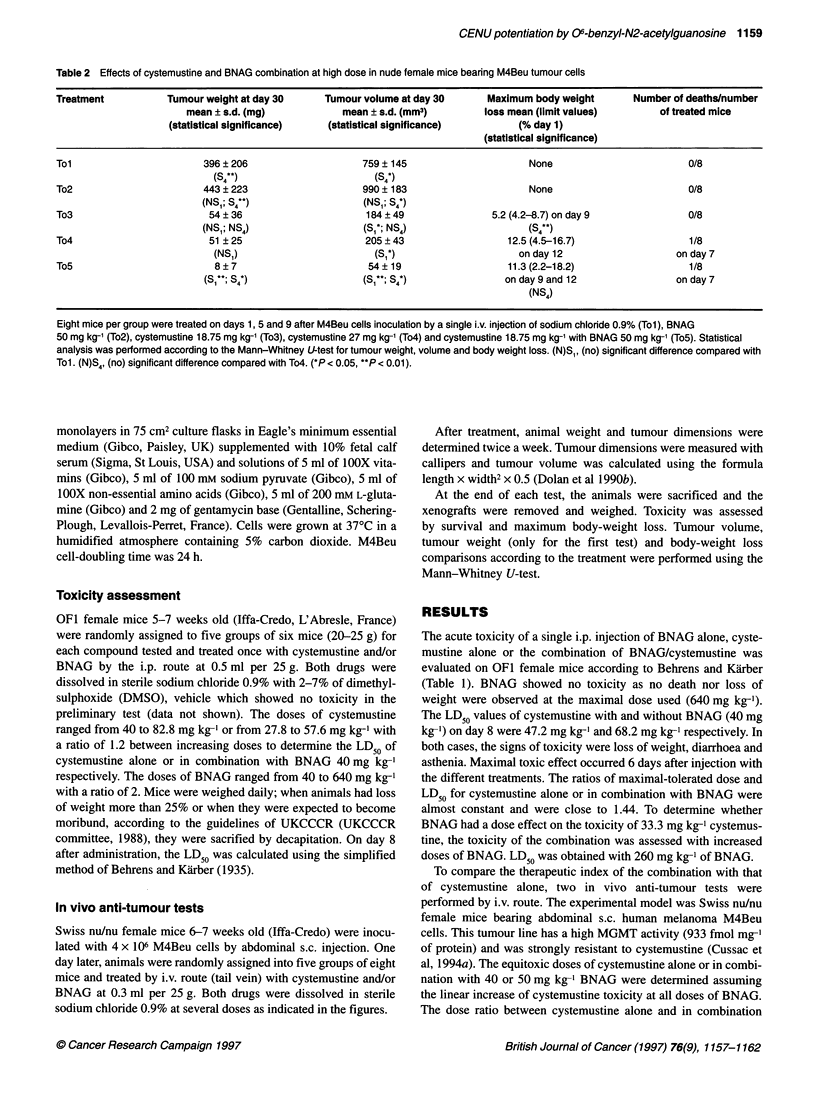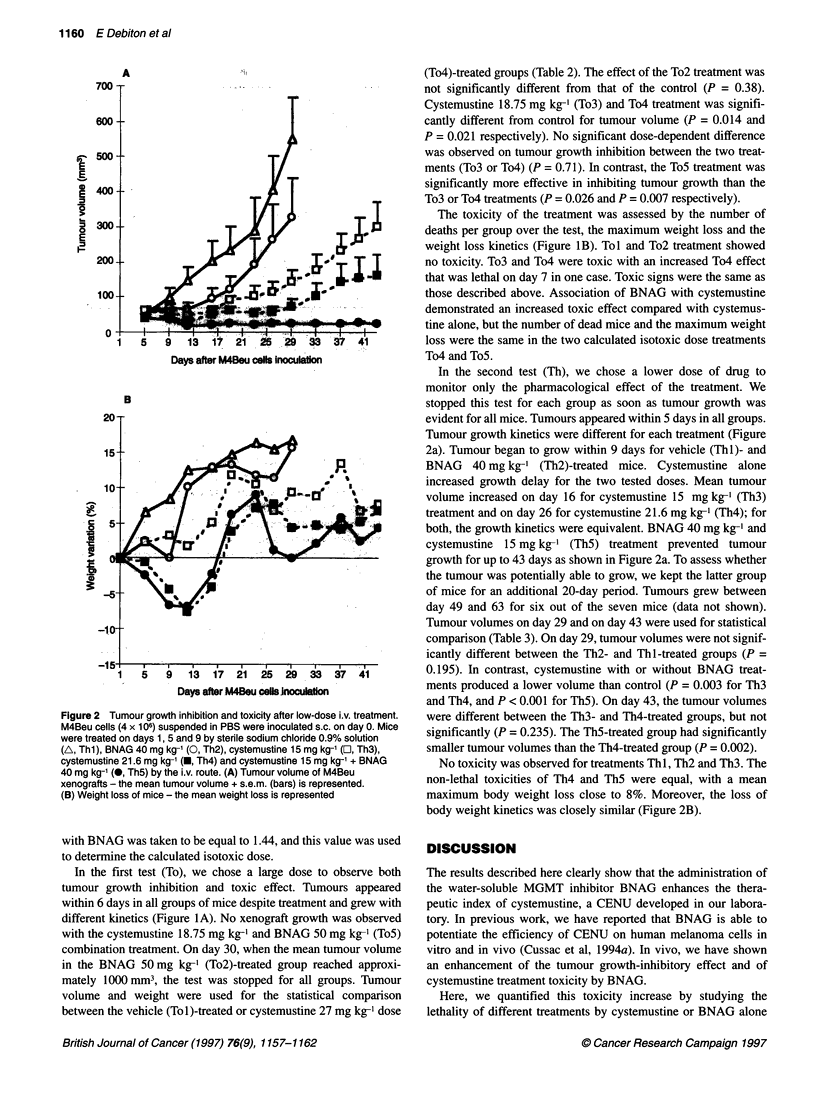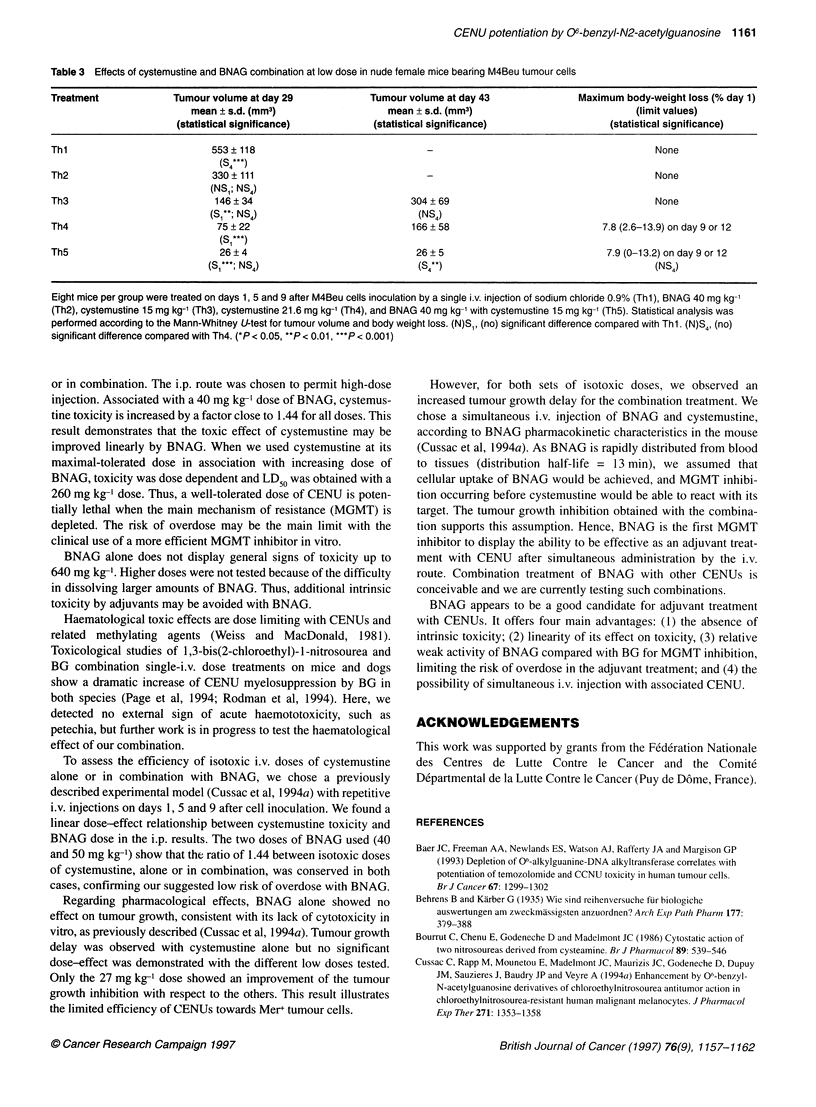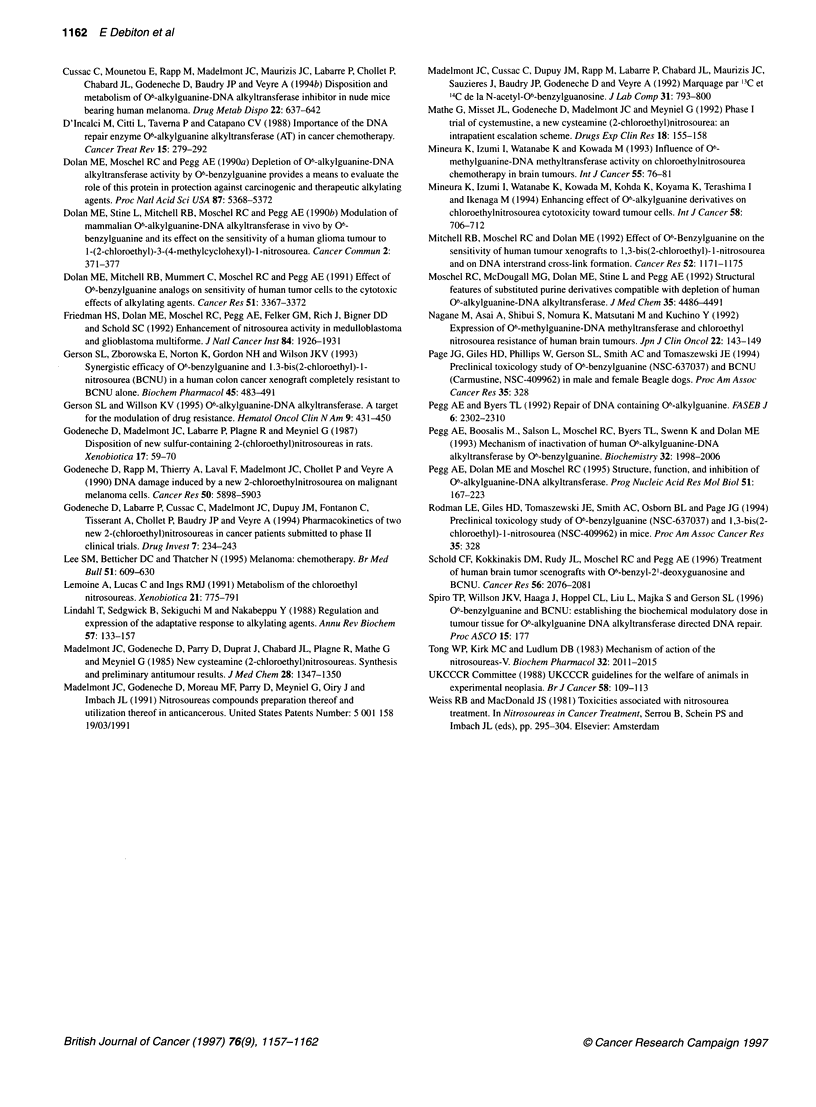# Enhancement by O6-benzyl-N2-acetylguanosine of N'-[2-chloroethyl]-N-[2-(methylsulphonyl)ethyl]-N'-nitrosourea therapeutic index on nude mice bearing resistant human melanoma.

**DOI:** 10.1038/bjc.1997.527

**Published:** 1997

**Authors:** E. Debiton, C. Cussac-Buchdhal, E. Mounetou, M. Rapp, J. M. Dupuy, J. C. Maurizis, A. Veyre, J. C. Madelmont

**Affiliations:** Institut National de la SantÃ© et de la Recherche MÃ©dicale U.71, Clermont-Ferrand, France.

## Abstract

The exposure of cells to O6-benzyl-N2-acetylguanosine (BNAG) and several guanine derivatives is known to reduce the activity of O6-alkylguanine-DNA alkyltransferase (MGMT) and to enhance the sensitivity of Mer+ (methyl enzyme repair positive) tumour cells to chloroethylnitrosoureas (CENUs) in vitro and in vivo. High water solubility and the pharmacokinetic properties of BNAG make it a candidate for simultaneous administration with CENUs by the i.v. route in human clinical use. In vivo we have shown previously that BNAG significantly increases the efficiency of N'-[2-chloroethyl]-N-[2-(methylsulphonyl)ethyl]-N'-nitrosourea (cystemustine) against M4Beu melanoma cells (Mer+) through its cytostatic activity by the i.p. route, but also increases its toxicity. To investigate the toxicity of BNAG and cystemustine when administered simultaneously in mice, we compared the maximum tolerated dose and LD50 doses of cystemustine alone or in combination with 40 mg kg(-1) BNAG by the i.p. route. The toxicity of cystemustine was enhanced by a factor of almost 1.44 when combined with BNAG. To compare the therapeutic index of cystemustine alone and the cystemustine/BNAG combination, pharmacological tests were carried out in nude mice bearing Mer+ M4Beu human melanoma cells. Isotoxic doses were calculated using the 1.44 ratio. The treatments were administered three times by the i.v. route on days 1, 5 and 9 after s.c. inoculation of tumour cells. Although the toxicities of the treatments were equal, BNAG strongly enhanced tumour growth inhibition. These results demonstrate the increase of the therapeutic index of cystemustine by BNAG and justify the use of BNAG to enhance nitrosourea efficiency in vivo by i.v. co-injection.


British Journal of Cancer (1997) 76(9), 1157–1162
© 1997 Cancer Research Campaign

### Enhancement by O$^6$-benzyl-N2-acetylguanosine of *N'*-[2-chloroethyl]-*N* [2-(methylsulphonyl)ethyl]-*N'*-nitrosourea therapeutic index on nude mice bearing resistant human melanoma

**E Debiton, C Cussac-Buchdhal, E Mounetou, M Rapp, JM Dupuy, J-C Maurizis, A Veyre and J-C Madelmont**

Institut National de la Santé et de la Recherche Médicale U.71, Rue Montalembert, 63005 Clermont-Ferrand Cedex, France

**Summary** The exposure of cells to O$^6$-benzyl-N2-acetylguanosine (BNAG) and several guanine derivatives is known to reduce the activity of O$^6$-alkylguanine-DNA alkyltransferase (MGMT) and to enhance the sensitivity of Mer$^+$ (methyl enzyme repair positive) tumour cells to chloroethylnitrosoureas (CENUs) in vitro and in vivo. High water solubility and the pharmacokinetic properties of BNAG make it a candidate for simultaneous administration with CENUs by the i.v. route in human clinical use. In vivo we have shown previously that BNAG significantly increases the efficiency of *N'*-[2-chloroethyl]-*N*-[2-(methylsulphonyl)ethyl]-*N'*-nitrosourea (cystemustine) against M4Beu melanoma cells (Mer$^+$) through its cytostatic activity by the i.p. route, but also increases its toxicity. To investigate the toxicity of BNAG and cystemustine when administered simultaneously in mice, we compared the maximum tolerated dose and $LD_{50}$ doses of cystemustine alone or in combination with 40 mg kg$^{-1}$ BNAG by the i.p. route. The toxicity of cystemustine was enhanced by a factor of almost 1.44 when combined with BNAG. To compare the therapeutic index of cystemustine alone and the cystemustine/BNAG combination, pharmacological tests were carried out in nude mice bearing Mer$^+$ M4Beu human melanoma cells. Isotoxic doses were calculated using the 1.44 ratio. The treatments were administered three times by the i.v. route on days 1, 5 and 9 after s.c. inoculation of tumour cells. Although the toxicities of the treatments were equal, BNAG strongly enhanced tumour growth inhibition. These results demonstrate the increase of the therapeutic index of cystemustine by BNAG and justify the use of BNAG to enhance nitrosourea efficiency in vivo by i.v. co-injection.

**Keywords:** O$^6$-benzyl-N2-acetylguanosine; O$^6$-alkylguanine-DNA alkyltransferase; *N'*-[2-chloroethyl]-*N*-[2-(methylsulphonyl)ethyl]-*N'*-nitrosourea; melanoma; therapeutic index

Chloroethylnitrosoureas (CENUs) are cancer chemotherapeutic drugs widely used in the treatment of several tumours, in particulars brain, systemic malignancies and melanomas. However, in most neoplasms, the response rate is relatively low after single-agent chemotherapy – about 20% in disseminated melanomas (Lee et al, 1995). CENUs are bialkylating agents and act through the formation of a chloroethyl cation that is able to react on several nucleophilic sites of the nucleic acids. The main cytotoxic lesion is O$^6$-chloroethylguanine, which is able spontaneously to form covalent cross-links with the complementary DNA strands (Tong et al, 1983; Lemoine et al, 1991). However, the suicidal DNA repair protein O$^6$-alkylguanine-DNA alkyltransferase (MGMT; EC 2.1.1.63) removes O$^6$-alkylguanine by accepting the alkyl group on the cystein residue of its active site (D'Incalci et al, 1988; Lindahl et al, 1988; Pegg and Byers, 1992). This protein is responsible for the chemoresistance of tumour cells towards CENUs and methylating agents (Nagane et al, 1992; Mineura et al, 1993; Gerson and Willson, 1995; Pegg et al, 1995). Its amount in the cell is inversely proportional to the DNA interstrand cross-links frequency and consequently to cell sensitivity to CENUs (Godeneche et al, 1990). The constitutive level of MGMT in mammals varies considerably from species to species but also from tissue to tissue within the same species (Pegg et al, 1995). A wide MGMT spectrum has been found in neoplasic tissues: cells that exhibit high MGMT levels have been designated Mer$^+$ (Mer for methyl enzyme repair) and those that are MGMT deficient as Mer$^-$. It has been shown previously that O$^6$-benzylguanine (BG) can significantly decrease the MGMT activity of Mer$^+$ cells through its ability to act as a substrate for the protein (Dolan et al, 1990*a*; Moschel et al, 1992; Pegg et al, 1993). This property has been used to increase the Mer$^+$ cell sensitivity to CENUs (Dolan et al, 1991; Baer et al, 1993; Mineura et al, 1994). In vivo studies conducted on nude mice bearing Mer$^+$ human colon or glioma tumours have shown that treatment with BG before administration of a bialkylating agent significantly inhibited tumour growth compared with animals treated with BG or a bialkylating agent alone (Dolan et al, 1990*b*; Friedman et al, 1992; Mitchell et al, 1992; Gerson et al, 1993). A phase I clinical trial of BG with carmustine is in progress (Spiro et al, 1996). Nevertheless, its clinical use can be problematic because of its very low water solubility, and new MGMT inhibitors have accordingly been developed that are more water soluble (Cussac et al, 1994*a*; Schold et al, 1996).

To address this problem, we have synthesized N2-acetylguanosine and deoxyguanosine derivatives that are benzylated on the O$^6$ position. The sugar moiety makes these species highly water soluble and the acetylation of the amine function should render pairing with the complementary base and the incorporation into

*Received 15 November 1996*
*Revised 7 April 1997*
*Accepted 15 April 1997*

*Correspondence to:* J-C Madelmont

**Table 1** Maximum-tolerated dose and $LD_{50}$ of cystemustine and/or BNAG on OF1 female mice at day 8 after treatments

| | Maximum-tolerated dose | $LD_{50}$ |
|---|---|---|
| BNAG[a] | 640[b] | |
| Cystemustine[a] | 48 | 68.2 |
| Cystemustine[a]+ 40 mg $kg^{-1}$ BNAG | 33.3 | 47.2 |
| Ratio | 1.44 | 1.45 |
| Cystemustine 33.3 mg $kg^{-1}$ + BNAG[a] | 80 | 260 |

[a]Variable doses. [b]Dose (mg $kg^{-1}$). Maximum-tolerated dose, highest dose administered without toxicity over the test. $LD_{50}$ was determined according to Behrens and Kärber.

**Figure 1** Tumour growth inhibition and toxicity after high-dose i.v. treatment. M4Beu cells (4 × $10^6$) suspended in PBS were inoculated s.c. on day 0. Mice were treated on days 1, 5 and 9 by sterile sodium chloride 0.9% solution (Δ, To1), BNAG 50 mg $kg^{-1}$ (○, To2), cystemustine 18.75 mg $kg^{-1}$ (□, To3), cystemustine 27 mg $kg^{-1}$ (■, To4) and cystemustine 18.75 mg $kg^{-1}$ + BNAG 50 mg $kg^{-1}$ (●, To5) by the i.v. route. (**A**) Tumour volume of M4Beu xenografts – the mean tumour volume + s.e.m. (bars) is represented. (**B**) Weight loss of mice after high-dose i.v. treatment – the mean weight loss is represented

DNA much more difficult. In previous work, BNAG and $O^6$-benzyl-N2-acetyldeoxyguanosine (BNAdG) have been tested for the potentiation of the cytotoxic effect of a new CENU, *N'*-[2-chloroethyl]-*N*-[2-(methylsulphony)ethyl]-*N'*-nitrosourea (cystemustine). This CENU was developed by us and is currently being used in melanoma and glioma clinical phase II trials of the European Organization for Research into the Treatment of Cancer (Madelmont et al, 1985; Bourrut et al, 1986; Mathe et al, 1992; Godeneche et al, 1994). We have shown that both BNAG and BNAdG significantly increased the cytotoxicity of cystemustine towards M4Beu human melanoma tumour cells ($Mer^+$) through the inhibition of MGMT. Preliminary in vivo anti-tumour tests on nude mice bearing M4Beu xenografts showed an increased inhibition of tumour growth by cystemustine in combination with BNAG but also an increased toxicity (Cussac et al, 1994*a*). To investigate interactive phenomena between cystemustine and BNAG, a pharmacokinetic study of BNAG i.v. bolus was performed using $^{14}C$ labelling (Madelmont et al, 1992; Cussac et al, 1994*b*). We showed that only 10% of the administered dose was metabolized and that the unchanged molecule had a rapid and wide distribution in all tissues except the central nervous system. Moreover, an enterohepatic cycle with a slow elimination rate extends the half-life of the molecule in the organism. From these pharmacokinetic characteristics, it is highly probable that MGMT inhibition, after an i.v. bolus, occurs promptly and is maintained for a long time after administration. Comparison with pharmacokinetic properties of cystemustine (Godeneche et al, 1987) indicates that a simultaneous i.v. injection of this CENU with BNAG should be the optimal administration schedule. In this work, our purpose is to compare the therapeutic index for the simultaneous administration of the BNAG/cystemustine combination with cystemustine alone in nude mice bearing resistant human melanoma. After measurement of the $LD_{50}$ of BNAG, cystemustine and the combination of BNAG/cystemustine, the effectiveness of treatments at isotoxic doses are compared.

### METHODS

### Drugs

Cystemustine was synthesized by usual procedures (Madelmont et al, 1991) and BNAG was synthesized by procedures previously described (Madelmont et al, 1992).

### Cells culture

M4Beu, a human melanoma cell line was derived from metastatic biopsy specimens and had been maintained in cell culture for almost 20 years at the Institute National de la Santé et de la Recherche Médicale, Unit 453, Centre Léon Bérard, Lyon, France (department of Dr JF Doré). Stock cell cultures were maintained as

© Cancer Research Campaign 1997

**Table 2** Effects of cystemustine and BNAG combination at high dose in nude female mice bearing M4Beu tumour cells

| Treatment | Tumour weight at day 30 mean ± s.d. (mg) (statistical significance) | Tumour volume at day 30 mean ± s.d. ($mm^3$) (statistical significance) | Maximum body weight loss mean (limit values) (% day 1) (statistical significance) | Number of deaths/number of treated mice |
|---|---|---|---|---|
| To1 | 396 ± 206 ($S_4$**) | 759 ± 145 ($S_4$*) | None | 0/8 |
| To2 | 443 ± 223 ($NS_1$; $S_4$**) | 990 ± 183 ($NS_1$; $S_4$*) | None | 0/8 |
| To3 | 54 ± 36 ($NS_1$; $NS_4$) | 184 ± 49 ($S_1$*; $NS_4$) | 5.2 (4.2–8.7) on day 9 ($S_4$**) | 0/8 |
| To4 | 51 ± 25 ($NS_1$) | 205 ± 43 ($S_1$*) | 12.5 (4.5–16.7) on day 12 | 1/8 on day 7 |
| To5 | 8 ± 7 ($S_1$**; $S_4$*) | 54 ± 19 ($S_1$**; $S_4$*) | 11.3 (2.2–18.2) on day 9 and 12 ($NS_4$) | 1/8 on day 7 |

Eight mice per group were treated on days 1, 5 and 9 after M4Beu cells inoculation by a single i.v. injection of sodium chloride 0.9% (To1), BNAG 50 mg $kg^{-1}$ (To2), cystemustine 18.75 mg $kg^{-1}$ (To3), cystemustine 27 mg $kg^{-1}$ (To4) and cystemustine 18.75 mg $kg^{-1}$ with BNAG 50 mg $kg^{-1}$ (To5). Statistical analysis was performed according to the Mann–Whitney *U*-test for tumour weight, volume and body weight loss. (N)$S_1$, (no) significant difference compared with To1. (N)$S_4$, (no) significant difference compared with To4. (*$P < 0.05$, **$P < 0.01$).

monolayers in 75 $cm^2$ culture flasks in Eagle's minimum essential medium (Gibco, Paisley, UK) supplemented with 10% fetal calf serum (Sigma, St Louis, USA) and solutions of 5 ml of 100X vitamins (Gibco), 5 ml of 100 mM sodium pyruvate (Gibco), 5 ml of 100X non-essential amino acids (Gibco), 5 ml of 200 mM L-glutamine (Gibco) and 2 mg of gentamycin base (Gentalline, Schering-Plough, Levallois-Perret, France). Cells were grown at 37°C in a humidified atmosphere containing 5% carbon dioxide. M4Beu cell-doubling time was 24 h.

### Toxicity assessment

OF1 female mice 5–7 weeks old (Iffa-Credo, L'Abresle, France) were randomly assigned to five groups of six mice (20–25 g) for each compound tested and treated once with cystemustine and/or BNAG by the i.p. route at 0.5 ml per 25 g. Both drugs were dissolved in sterile sodium chloride 0.9% with 2–7% of dimethylsulphoxide (DMSO), vehicle which showed no toxicity in the preliminary test (data not shown). The doses of cystemustine ranged from 40 to 82.8 mg $kg^{-1}$ or from 27.8 to 57.6 mg $kg^{-1}$ with a ratio of 1.2 between increasing doses to determine the $LD_{50}$ of cystemustine alone or in combination with BNAG 40 mg $kg^{-1}$ respectively. The doses of BNAG ranged from 40 to 640 mg $kg^{-1}$ with a ratio of 2. Mice were weighed daily; when animals had loss of weight more than 25% or when they were expected to become moribund, according to the guidelines of UKCCCR (UKCCCR committee, 1988), they were sacrified by decapitation. On day 8 after administration, the $LD_{50}$ was calculated using the simplified method of Behrens and Kärber (1935).

### In vivo anti-tumour tests

Swiss nu/nu female mice 6–7 weeks old (Iffa-Credo) were inoculated with 4 × $10^6$ M4Beu cells by abdominal s.c. injection. One day later, animals were randomly assigned into five groups of eight mice and treated by i.v. route (tail vein) with cystemustine and/or BNAG at 0.3 ml per 25 g. Both drugs were dissolved in sterile sodium chloride 0.9% at several doses as indicated in the figures.

After treatment, animal weight and tumour dimensions were determined twice a week. Tumour dimensions were measured with callipers and tumour volume was calculated using the formula length × width$^2$ × 0.5 (Dolan et al 1990*b*).

At the end of each test, the animals were sacrificed and the xenografts were removed and weighed. Toxicity was assessed by survival and maximum body-weight loss. Tumour volume, tumour weight (only for the first test) and body-weight loss comparisons according to the treatment were performed using the Mann–Whitney *U*-test.

### RESULTS

The acute toxicity of a single i.p. injection of BNAG alone, cystemustine alone or the combination of BNAG/cystemustine was evaluated on OF1 female mice according to Behrens and Kärber (Table 1). BNAG showed no toxicity as no death nor loss of weight were observed at the maximal dose used (640 mg $kg^{-1}$). The $LD_{50}$ values of cystemustine with and without BNAG (40 mg $kg^{-1}$) on day 8 were 47.2 mg $kg^{-1}$ and 68.2 mg $kg^{-1}$ respectively. In both cases, the signs of toxicity were loss of weight, diarrhoea and asthenia. Maximal toxic effect occurred 6 days after injection with the different treatments. The ratios of maximal-tolerated dose and $LD_{50}$ for cystemustine alone or in combination with BNAG were almost constant and were close to 1.44. To determine whether BNAG had a dose effect on the toxicity of 33.3 mg $kg^{-1}$ cystemustine, the toxicity of the combination was assessed with increased doses of BNAG. $LD_{50}$ was obtained with 260 mg $kg^{-1}$ of BNAG.

To compare the therapeutic index of the combination with that of cystemustine alone, two in vivo anti-tumour tests were performed by i.v. route. The experimental model was Swiss nu/nu female mice bearing abdominal s.c. human melanoma M4Beu cells. This tumour line has a high MGMT activity (933 fmol $mg^{-1}$ of protein) and was strongly resistant to cystemustine (Cussac et al, 1994*a*). The equitoxic doses of cystemustine alone or in combination with 40 or 50 mg $kg^{-1}$ BNAG were determined assuming the linear increase of cystemustine toxicity at all doses of BNAG. The dose ratio between cystemustine alone and in combination

© Cancer Research Campaign 1997

**Figure 2** Tumour growth inhibition and toxicity after low-dose i.v. treatment. M4Beu cells ($4 \times 10^6$) suspended in PBS were inoculated s.c. on day 0. Mice were treated on days 1, 5 and 9 by sterile sodium chloride 0.9% solution (△, Th1), BNAG 40 mg kg$^{-1}$ (○, Th2), cystemustine 15 mg kg$^{-1}$ (□, Th3), cystemustine 21.6 mg kg$^{-1}$ (■, Th4) and cystemustine 15 mg kg$^{-1}$ + BNAG 40 mg kg$^{-1}$ (●, Th5) by the i.v. route. (**A**) Tumour volume of M4Beu xenografts – the mean tumour volume + s.e.m. (bars) is represented. (**B**) Weight loss of mice – the mean weight loss is represented with BNAG was taken to be equal to 1.44, and this value was used to determine the calculated isotoxic dose.

In the first test (To), we chose a large dose to observe both tumour growth inhibition and toxic effect. Tumours appeared within 6 days in all groups of mice despite treatment and grew with different kinetics (Figure 1A). No xenograft growth was observed with the cystemustine 18.75 mg kg$^{-1}$ and BNAG 50 mg kg$^{-1}$ (To5) combination treatment. On day 30, when the mean tumour volume in the BNAG 50 mg kg$^{-1}$ (To2)-treated group reached approximately 1000 mm$^3$, the test was stopped for all groups. Tumour volume and weight were used for the statistical comparison between the vehicle (To1)-treated or cystemustine 27 mg kg$^{-1}$ dose (To4)-treated groups (Table 2). The effect of the To2 treatment was not significantly different from that of the control ($P$ = 0.38). Cystemustine 18.75 mg kg$^{-1}$ (To3) and To4 treatment was significantly different from control for tumour volume ($P$ = 0.014 and $P$ = 0.021 respectively). No significant dose-dependent difference was observed on tumour growth inhibition between the two treatments (To3 or To4) ($P$ = 0.71). In contrast, the To5 treatment was significantly more effective in inhibiting tumour growth than the To3 or To4 treatments ($P$ = 0.026 and $P$ = 0.007 respectively).

The toxicity of the treatment was assessed by the number of deaths per group over the test, the maximum weight loss and the weight loss kinetics (Figure 1B). To1 and To2 treatment showed no toxicity. To3 and To4 were toxic with an increased To4 effect that was lethal on day 7 in one case. Toxic signs were the same as those described above. Association of BNAG with cystemustine demonstrated an increased toxic effect compared with cystemustine alone, but the number of dead mice and the maximum weight loss were the same in the two calculated isotoxic dose treatments To4 and To5.

In the second test (Th), we chose a lower dose of drug to monitor only the pharmacological effect of the treatment. We stopped this test for each group as soon as tumour growth was evident for all mice. Tumours appeared within 5 days in all groups. Tumour growth kinetics were different for each treatment (Figure 2a). Tumour began to grow within 9 days for vehicle (Th1)- and BNAG 40 mg kg$^{-1}$ (Th2)-treated mice. Cystemustine alone increased growth delay for the two tested doses. Mean tumour volume increased on day 16 for cystemustine 15 mg kg$^{-1}$ (Th3) treatment and on day 26 for cystemustine 21.6 mg kg$^{-1}$ (Th4); for both, the growth kinetics were equivalent. BNAG 40 mg kg$^{-1}$ and cystemustine 15 mg kg$^{-1}$ (Th5) treatment prevented tumour growth for up to 43 days as shown in Figure 2a. To assess whether the tumour was potentially able to grow, we kept the latter group of mice for an additional 20-day period. Tumours grew between day 49 and 63 for six out of the seven mice (data not shown). Tumour volumes on day 29 and on day 43 were used for statistical comparison (Table 3). On day 29, tumour volumes were not significantly different between the Th2- and Th1-treated groups ($P$ = 0.195). In contrast, cystemustine with or without BNAG treatments produced a lower volume than control ($P$ = 0.003 for Th3 and Th4, and $P < 0.001$ for Th5). On day 43, the tumour volumes were different between the Th3- and Th4-treated groups, but not significantly ($P$ = 0.235). The Th5-treated group had significantly smaller tumour volumes than the Th4-treated group ($P$ = 0.002).

No toxicity was observed for treatments Th1, Th2 and Th3. The non-lethal toxicities of Th4 and Th5 were equal, with a mean maximum body weight loss close to 8%. Moreover, the loss of body weight kinetics was closely similar (Figure 2B).

### DISCUSSION

The results described here clearly show that the administration of the water-soluble MGMT inhibitor BNAG enhances the therapeutic index of cystemustine, a CENU developed in our laboratory. In previous work, we have reported that BNAG is able to potentiate the efficiency of CENU on human melanoma cells in vitro and in vivo (Cussac et al, 1994*a*). In vivo, we have shown an enhancement of the tumour growth-inhibitory effect and of cystemustine treatment toxicity by BNAG.

Here, we quantified this toxicity increase by studying the lethality of different treatments by cystemustine or BNAG alone

 © Cancer Research Campaign 1997

**Table 3** Effects of cystemustine and BNAG combination at low dose in nude female mice bearing M4Beu tumour cells

| Treatment | Tumour volume at day 29 mean ± s.d. (mm³) (statistical significance) | Tumour volume at day 43 mean ± s.d. (mm³) (statistical significance) | Maximum body-weight loss (% day 1) (limit values) (statistical significance) |
|---|---|---|---|
| Th1 | 553 ± 118 ($S_4$***) | – | None |
| Th2 | 330 ± 111 ($NS_1$; $NS_4$) | – | None |
| Th3 | 146 ± 34 ($S_1$**; $NS_4$) | 304 ± 69 ($NS_4$) | None |
| Th4 | 75 ± 22 ($S_1$***) | 166 ± 58 | 7.8 (2.6–13.9) on day 9 or 12 |
| Th5 | 26 ± 4 ($S_1$***; $NS_4$) | 26 ± 5 ($S_4$**) | 7.9 (0–13.2) on day 9 or 12 ($NS_4$) |

Eight mice per group were treated on days 1, 5 and 9 after M4Beu cells inoculation by a single i.v. injection of sodium chloride 0.9% (Th1), BNAG 40 mg kg$^{-1}$ (Th2), cystemustine 15 mg kg$^{-1}$ (Th3), cystemustine 21.6 mg kg$^{-1}$ (Th4), and BNAG 40 mg kg$^{-1}$ with cystemustine 15 mg kg$^{-1}$ (Th5). Statistical analysis was performed according to the Mann-Whitney *U*-test for tumour volume and body weight loss. (N)$S_1$, (no) significant difference compared with Th1. (N)$S_4$, (no) significant difference compared with Th4. (**P* < 0.05, ***P* < 0.01, ****P* < 0.001)

or in combination. The i.p. route was chosen to permit high-dose injection. Associated with a 40 mg kg$^{-1}$ dose of BNAG, cystemustine toxicity is increased by a factor close to 1.44 for all doses. This result demonstrates that the toxic effect of cystemustine may be improved linearly by BNAG. When we used cystemustine at its maximal-tolerated dose in association with increasing dose of BNAG, toxicity was dose dependent and $LD_{50}$ was obtained with a 260 mg kg$^{-1}$ dose. Thus, a well-tolerated dose of CENU is potentially lethal when the main mechanism of resistance (MGMT) is depleted. The risk of overdose may be the main limit with the clinical use of a more efficient MGMT inhibitor in vitro.

BNAG alone does not display general signs of toxicity up to 640 mg kg$^{-1}$. Higher doses were not tested because of the difficulty in dissolving larger amounts of BNAG. Thus, additional intrinsic toxicity by adjuvants may be avoided with BNAG.

Haematological toxic effects are dose limiting with CENUs and related methylating agents (Weiss and MacDonald, 1981). Toxicological studies of 1,3-bis(2-chloroethyl)-1-nitrosourea and BG combination single-i.v. dose treatments on mice and dogs show a dramatic increase of CENU myelosuppression by BG in both species (Page et al, 1994; Rodman et al, 1994). Here, we detected no external sign of acute haemototoxicity, such as petechia, but further work is in progress to test the haematological effect of our combination.

To assess the efficiency of isotoxic i.v. doses of cystemustine alone or in combination with BNAG, we chose a previously described experimental model (Cussac et al, 1994*a*) with repetitive i.v. injections on days 1, 5 and 9 after cell inoculation. We found a linear dose–effect relationship between cystemustine toxicity and BNAG dose in the i.p. results. The two doses of BNAG used (40 and 50 mg kg$^{-1}$) show that the ratio of 1.44 between isotoxic doses of cystemustine, alone or in combination, was conserved in both cases, confirming our suggested low risk of overdose with BNAG.

Regarding pharmacological effects, BNAG alone showed no effect on tumour growth, consistent with its lack of cytotoxicity in vitro, as previously described (Cussac et al, 1994*a*). Tumour growth delay was observed with cystemustine alone but no significant dose–effect was demonstrated with the different low doses tested. Only the 27 mg kg$^{-1}$ dose showed an improvement of the tumour growth inhibition with respect to the others. This result illustrates the limited efficiency of CENUs towards $Mer^+$ tumour cells.

However, for both sets of isotoxic doses, we observed an increased tumour growth delay for the combination treatment. We chose a simultaneous i.v. injection of BNAG and cystemustine, according to BNAG pharmacokinetic characteristics in the mouse (Cussac et al, 1994*a*). As BNAG is rapidly distributed from blood to tissues (distribution half-life = 13 min), we assumed that cellular uptake of BNAG would be achieved, and MGMT inhibition occurring before cystemustine would be able to react with its target. The tumour growth inhibition obtained with the combination supports this assumption. Hence, BNAG is the first MGMT inhibitor to display the ability to be effective as an adjuvant treatment with CENU after simultaneous administration by the i.v. route. Combination treatment of BNAG with other CENUs is conceivable and we are currently testing such combinations.

BNAG appears to be a good candidate for adjuvant treatment with CENUs. It offers four main advantages: (1) the absence of intrinsic toxicity; (2) linearity of its effect on toxicity, (3) relative weak activity of BNAG compared with BG for MGMT inhibition, limiting the risk of overdose in the adjuvant treatment; and (4) the possibility of simultaneous i.v. injection with associated CENU.

### ACKNOWLEDGEMENTS

This work was supported by grants from the Fédération Nationale des Centres de Lutte Contre le Cancer and the Comité Départmental de la Lutte Contre le Cancer (Puy de Dôme, France).

### REFERENCES

Baer JC, Freeman AA, Newlands ES, Watson AJ, Rafferty JA and Margison GP (1993) Depletion of O$^6$-alkylguanine-DNA alkyltransferase correlates with potentiation of temozolomide and CCNU toxicity in human tumour cells. *Br J Cancer* **67**: 1299–1302

Behrens B and Kärber G (1935) Wie sind reihenversuche für biologiche auswertungen am zweckmässigsten anzuordnen? *Arch Exp Path Pharm* **177**: 379–388

Bourrut C, Chenu E, Godeneche D and Madelmont JC (1986) Cytostatic action of two nitrosoureas derived from cysteamine. *Br J Pharmacol* **89**: 539–546

Cussac C, Rapp M, Mounetou E, Madelmont JC, Maurizis JC, Godeneche D, Dupuy JM, Sauzieres J, Baudry JP and Veyre A (1994*a*) Enhancement by O$^6$-benzyl-N-acetylguanosine derivatives of chloroethylnitrosourea antitumor action in chloroethylnitrosourea-resistant human malignant melanocytes. *J Pharmacol Exp Ther* **271**: 1353–1358

Cussac C, Mounetou E, Rapp M, Madelmont JC, Maurizis JC, Labarre P, Chollet P, Chabard JL, Godeneche D, Baudry JP and Veyre A (1994*b*) Disposition and metabolism of O$^6$-alkylguanine-DNA alkyltransferase inhibitor in nude mice bearing human melanoma. *Drug Metab Dispo* **22**: 637–642

D'Incalci M, Citti L, Taverna P and Catapano CV (1988) Importance of the DNA repair enzyme O$^6$-alkylguanine alkyltransferase (AT) in cancer chemotherapy. *Cancer Treat Rev* **15**: 279–292

Dolan ME, Moschel RC and Pegg AE (1990*a*) Depletion of O$^6$-alkylguanine-DNA alkyltransferase activity by O$^6$-benzylguanine provides a means to evaluate the role of this protein in protection against carcinogenic and therapeutic alkylating agents. *Proc Natl Acid Sci USA* **87**: 5368–5372

Dolan ME, Stine L, Mitchell RB, Moschel RC and Pegg AE (1990*b*) Modulation of mammalian O$^6$-alkylguanine-DNA alkyltransferase in vivo by O$^6$-benzylguanine and its effect on the sensitivity of a human glioma tumour to 1-(2-chloroethyl)-3-(4-methylcyclohexyl)-1-nitrosourea. *Cancer Commun* **2**: 371–377

Dolan ME, Mitchell RB, Mummert C, Moschel RC and Pegg AE (1991) Effect of O$^6$-benzylguanine analogs on sensitivity of human tumor cells to the cytotoxic effects of alkylating agents. *Cancer Res* **51**: 3367–3372

Friedman HS, Dolan ME, Moschel RC, Pegg AE, Felker GM, Rich J, Bigner DD and Schold SC (1992) Enhancement of nitrosourea activity in medulloblastoma and glioblastoma multiforme. *J Natl Cancer Inst* **84**: 1926–1931

Gerson SL, Zborowska E, Norton K, Gordon NH and Wilson JKV (1993) Synergistic efficacy of O$^6$-benzylguanine and 1.3-bis(2-chloroethyl)-1-nitrosourea (BCNU) in a human colon cancer xenograft completely resistant to BCNU alone. *Biochem Pharmacol* **45**: 483–491

Gerson SL and Willson KV (1995) O$^6$-alkylguanine-DNA alkyltransferase. A target for the modulation of drug resistance. *Hematol Oncol Clin N Am* **9**: 431–450

Godeneche D, Madelmont JC, Labarre P, Plagne R and Meyniel G (1987) Disposition of new sulfur-containing 2-(chloroethyl)nitrosoureas in rats. *Xenobiotica* **17**: 59–70

Godeneche D, Rapp M, Thierry A, Laval F, Madelmont JC, Chollet P and Veyre A (1990) DNA damage induced by a new 2-chloroethylnitrosourea on malignant melanoma cells. *Cancer Res* **50**: 5898–5903

Godeneche D, Labarre P, Cussac C, Madelmont JC, Dupuy JM, Fontanon C, Tisserant A, Chollet P, Baudry JP and Veyre A (1994) Pharmacokinetics of two new 2-(chloroethyl)nitrosoureas in cancer patients submitted to phase II clinical trials. *Drug Invest* **7**: 234–243

Lee SM, Betticher DC and Thatcher N (1995) Melanoma: chemotherapy. *Br Med Bull* **51**: 609–630

Lemoine A, Lucas C and Ings RMJ (1991) Metabolism of the chloroethyl nitrosoureas. *Xenobiotica* **21**: 775–791

Lindahl T, Sedgwick B, Sekiguchi M and Nakabeppu Y (1988) Regulation and expression of the adaptative response to alkylating agents. *Annu Rev Biochem* **57**: 133–157

Madelmont JC, Godeneche D, Parry D, Duprat J, Chabard JL, Plagne R, Mathe G and Meyniel G (1985) New cysteamine (2-chloroethyl)nitrosoureas. Synthesis and preliminary antitumour results. *J Med Chem* **28**: 1347–1350

Madelmont JC, Godeneche D, Moreau MF, Parry D, Meyniel G, Oiry J and Imbach JL (1991) Nitrosoureas compounds preparation thereof and utilization thereof in anticancerous. United States Patents Number: 5 001 158 19/03/1991

Madelmont JC, Cussac C, Dupuy JM, Rapp M, Labarre P, Chabard JL, Maurizis JC, Sauzieres J, Baudry JP, Godeneche D and Veyre A (1992) Marquage par $^{13}$C et $^{14}$C de la N-acetyl-O$^6$-benzylguanosine. *J Lab Comp* **31**: 793–800

Mathe G, Misset JL, Godeneche D, Madelmont JC and Meyniel G (1992) Phase I trial of cystemustine, a new cysteamine (2-chloroethyl)nitrosourea: an intrapatient escalation scheme. *Drugs Exp Clin Res* **18**: 155–158

Mineura K, Izumi I, Watanabe K and Kowada M (1993) Influence of O$^6$-methylguanine-DNA methyltransferase activity on chloroethylnitrosourea chemotherapy in brain tumours. *Int J Cancer* **55**: 76–81

Mineura K, Izumi I, Watanabe K, Kowada M, Kohda K, Koyama K, Terashima I and Ikenaga M (1994) Enhancing effect of O$^6$-alkylguanine derivatives on chloroethylnitrosourea cytotoxicity toward tumour cells. *Int J Cancer* **58**: 706–712

Mitchell RB, Moschel RC and Dolan ME (1992) Effect of O$^6$-Benzylguanine on the sensitivity of human tumour xenografts to 1,3-bis(2-chloroethyl)-1-nitrosourea and on DNA interstrand cross-link formation. *Cancer Res* **52**: 1171–1175

Moschel RC, McDougall MG, Dolan ME, Stine L and Pegg AE (1992) Structural features of substituted purine derivatives compatible with depletion of human O$^6$-alkylguanine-DNA alkyltransferase. *J Med Chem* **35**: 4486–4491

Nagane M, Asai A, Shibui S, Nomura K, Matsutani M and Kuchino Y (1992) Expression of O$^6$-methylguanine-DNA methyltransferase and chloroethyl nitrosourea resistance of human brain tumours. *Jpn J Clin Oncol* **22**: 143–149

Page JG, Giles HD, Phillips W, Gerson SL, Smith AC and Tomaszewski JE (1994) Preclinical toxicology study of O$^6$-benzylguanine (NSC-637037) and BCNU (Carmustine, NSC-409962) in male and female Beagle dogs. *Proc Am Assoc Cancer Res* **35**: 328

Pegg AE and Byers TL (1992) Repair of DNA containing O$^6$-alkylguanine. *FASEB J* **6**: 2302–2310

Pegg AE, Boosalis M., Salson L, Moschel RC, Byers TL, Swenn K and Dolan ME (1993) Mechanism of inactivation of human O$^6$-alkylguanine-DNA alkyltransferase by O$^6$-benzylguanine. *Biochemistry* **32**: 1998–2006

Pegg AE, Dolan ME and Moschel RC (1995) Structure, function, and inhibition of O$^6$-alkylguanine-DNA alkyltransferase. *Prog Nucleic Acid Res Mol Biol* **51**: 167–223

Rodman LE, Giles HD, Tomaszewski JE, Smith AC, Osborn BL and Page JG (1994) Preclinical toxicology study of O$^6$-benzylguanine (NSC-637037) and 1,3-bis(2-chloroethyl)-1-nitrosourea (NSC-409962) in mice. *Proc Am Assoc Cancer Res* **35**: 328

Schold CF, Kokkinakis DM, Rudy JL, Moschel RC and Pegg AE (1996) Treatment of human brain tumor scenografts with O$^6$-benzyl-2$^1$-deoxyguanosine and BCNU. *Cancer Res* **56**: 2076–2081

Spiro TP, Willson JKV, Haaga J, Hoppel CL, Liu L, Majka S and Gerson SL (1996) O$^6$-benzylguanine and BCNU: establishing the biochemical modulatory dose in tumour tissue for O$^6$-alkylguanine DNA alkyltransferase directed DNA repair. *Proc ASCO* **15**: 177

Tong WP, Kirk MC and Ludlum DB (1983) Mechanism of action of the nitrosoureas-V. *Biochem Pharmacol* **32**: 2011–2015

UKCCCR Committee (1988) UKCCCR guidelines for the welfare of animals in experimental neoplasia. *Br J Cancer* **58**: 109–113

Weiss RB and MacDonald JS (1981) Toxicities associated with nitrosourea treatment. In *Nitrosoureas in Cancer Treatment*, Serrou B, Schein PS and Imbach JL (eds), pp. 295–304. Elsevier: Amsterdam

© Cancer Research Campaign 1997